# Deep Learning for Perfusion Cerebral Blood Flow (CBF) and Volume (CBV) Predictions and Diagnostics

**DOI:** 10.1007/s10439-024-03471-7

**Published:** 2024-02-24

**Authors:** Salmonn Talebi, Siyu Gai, Aaron Sossin, Vivian Zhu, Elizabeth Tong, Mohammad R. K. Mofrad

**Affiliations:** 1 Departments of Bioengineering and Mechanical Engineering, University of California, 208A Stanley Hall #1762, Berkeley, CA 94720-1762 USA; 2 Departments of Electrical Engineering and Computer Science, University of California, Berkeley, California USA; 3 Department of Bioinformatics, Stanford School of Medicine, Stanford University, Stanford, California USA; 4 Department of Radiology, Stanford School of Medicine, Stanford University, 725 Welch Rd Rm 1860, Palo Alto, Stanford, CA 94304 USA

**Keywords:** Deep learning, Perfusion, Cerebral blood volume, Cerebral blood flow, Magnetic resonance imaging

## Abstract

Dynamic susceptibility contrast magnetic resonance perfusion (DSC-MRP) is a non-invasive imaging technique for hemodynamic measurements. Various perfusion parameters, such as cerebral blood volume (CBV) and cerebral blood flow (CBF), can be derived from DSC-MRP, hence this non-invasive imaging protocol is widely used clinically for the diagnosis and assessment of intracranial pathologies. Currently, most institutions use commercially available software to compute the perfusion parametric maps. However, these conventional methods often have limitations, such as being time-consuming and sensitive to user input, which can lead to inconsistent results; this highlights the need for a more robust and efficient approach like deep learning. Using the relative cerebral blood volume (rCBV) and relative cerebral blood flow (rCBF) perfusion maps generated by FDA-approved software, we trained a multistage deep learning model. The model, featuring a combination of a 1D convolutional neural network (CNN) and a 2D U-Net encoder-decoder network, processes each 4D MRP dataset by integrating temporal and spatial features of the brain for voxel-wise perfusion parameters prediction. An auxiliary model, with similar architecture, but trained with truncated datasets that had fewer time-points, was designed to explore the contribution of temporal features. Both qualitatively and quantitatively evaluated, deep learning-generated rCBV and rCBF maps showcased effective integration of temporal and spatial data, producing comprehensive predictions for the entire brain volume. Our deep learning model provides a robust and efficient approach for calculating perfusion parameters, demonstrating comparable performance to FDA-approved commercial software, and potentially mitigating the challenges inherent to traditional techniques.

## Introduction

Going beyond anatomic imaging, magnetic resonance perfusion (MRP) imaging can provide hemodynamic information of the brain. MRP is commonly used in a wide range of clinical applications, including the assessment of stroke, tumor grading, differentiating tumor mimics, treatment decisions, etc. [1]. Dynamic susceptibility contrast (DSC) is a common non-invasive MRP technique. In DSC, dynamic T2*-weighted images are rapidly acquired after injecting a small gadolinium bolus, to capture the concentration of gadolinium within the brain every 1 to 2 s. The signal intensity-versus-time curve at each voxel is inversely related to the concentration of gadolinium, which in turn is proportional to the amount of blood in that voxel. Various perfusion parameters, such as relative cerebral blood volume (rCBV) and cerebral blood flow (rCBF), can be derived from DSC images. DSC computes perfusion parameters by using a dynamic first-pass model to examine the hemodynamic relationships between the arterial, brain tissue, and the venous compartments. The dynamic first-pass model assumes that the tracer is not diffusible and not absorbed by the brain tissue. Under these assumptions, according to the conservation-of-mass principle, this total amount of blood reaching the brain tissue must be equal to the amount leaving the brain tissue. Tracer kinetic theory states that if input and the output waveforms of a voxel are known, then the volume of distribution (blood volume) and the flow rate (blood flow) can be determined by mathematical models [2–4].

Commercial software packages are often utilized to compute the perfusion maps. The underlying algorithms of these software tools are often proprietary, but most of them are based on a 2-compartment perfusion model. Within the premise of this model, the generation of the perfusion parameters requires two vascular references—arterial input function (AIF) and venous output function (VOF). In general, cerebral perfusion parameters are dependent with a number of factors including cardiac output, caliber of the vasculature, bolus size, and injection rate. This dependence is minimized by deconvolving the arterial input function (AIF) from the tissue intensity-time waveforms. The derived perfusion parametric maps may differ according to the waveform of the AIF. The lack of consensus in the optimal choice of the AIF further impairs the reliability of the parametric maps [5–8]. Similarly, the VOF is used to compensate for partial volume effect in the AIF. Again, the choice of VOF will introduce variability in the parametric maps. It is beneficial to find an efficient method to generate perfusion parametric maps that are reproducible and reliable.

Deep learning (DL) is a rapidly emerging technique which has been increasingly applied to various fields in medicine such as cardiology [9], radiology [10–12], ophthalmology [13, 14], dermatology [15, 16], and pathology [17, 18]. Deep learning algorithms may learn from examples and respond to new inputs based on their prior training [19]. Deep learning may provide a means to generate perfusion parametric maps that are reproducible and reliable and can facilitate the assessment of stroke, tumor grading, differentiating tumor mimics, treatment decisions, etc. The objective of the present study is to use deep learning neural networks to produce perfusion parametric maps with the explicit choice of an arterial input function (AIF). This will eliminate the influence of AIF and produce perfusion parameters that reflect the (patho)physiological hemodynamics more reliably. Using perfusion maps generated by an FDA-approved software as ground truth, we trained DL models to predict rCBV and rCBF maps in patients with conditions associated with abnormal perfusion such as epilepsy [20], tumor [21], infectious or inflammatory, etc.

The simultaneous spatial and time-resolved nature of DSC complicated this task of predicting parametric maps. Conventional methods, such as using a convolutional neural network (CNN), on volumetric data can be memory intensive and challenging for the model to learn. In this study, we propose a two-encoder approach that first encodes the one-dimensional waveforms and then encodes the spatial information using a convolutional network with the U-Net architecture. This method significantly speeds up training and reduces memory requirements.

## Materials and Methods

This retrospective study was conducted with the approval of the Stanford Institutional Review Board (IRB) and under a waiver of informed consent. The study was approved for collaboration between Stanford University and the University of California, Berkeley. We retrospectively collected patients who underwent perfusion MRI brain imaging for clinical work-up between and found to have abnormal elevated rCBV or rCBF. Using keywords ‘hyperperfusion,’ ‘hypoperfusion,’ and ‘abnormal perfusion’ to search the radiologic imaging archive resulted in 158 patients. An additional 22 patients with no perfusion abnormality were collected using keywords ‘no perfusion abnormality’ or ‘normal perfusion.’ Out of the 180 patients, 21 studies were found to have motion artifacts or suboptimally generated perfusion maps and were excluded from the dataset. This ensures the model is not trained on invalid ground truths given by the commercial software. The underlying diagnoses of the 159 patients (74 females and 85 males, mean age 69 ± 11 years, range 58–80 years) are summarized in Table 1.

**Table 1:** Summary of the underlying clinical diagnoses of the subjects used in this study

Demographics	
Age	
Mean	69 years old
Range	58 to 80 years old
Gender:	74 females, 85 males
Diagnosis	Number of Cases
Normal	22
Brain mass	70
Seizure	14
Stroke	38
Infection	15
Total	159

59 patients (74 females and 85 males, mean age 69 ± 11 years, range 58–80 years)

### MR Imaging Acquisition Protocol

Brain MRI was performed on a 3.0 *T* GE MRI scanner using an 8 channel GE HR brain coil (GE Healthcare, Milwaukee, Wisconsin). DSC-MRP was performed using a dynamic susceptibility contrast technique following the intravenous administration of Multihance (Bracco, Milan, Italy) into an antecubital vein at a rate of 4.0 ml/s using a power injector. DSC parameters were as follows: TR = 1800 ms, TE = 35 ms; flip angle 80°, spatial resolution = 128 × 128, and slice thickness of 5 mm. A total of 60 whole brain volumes were acquired over approximately 2–3 minutes. Relative cerebral blood flow (rCBF) and relative cerebral blood volume (rCBV) were generated by the RAPID software (iSchemaView, Menlo Park, CA) [22].

### Data Preprocessing

The MRP datasets were divided into 131 patients for training and 28 for testing. There were 60 time-samples for each subject, with one whole brain volume for each time-time-sample. The number of slices needed to cover the entire brain of each subject varied from 18 to 22 slices. Both training and validation datasets consist of a similar mix of patients within each of the diagnostic categories.

Raw perfusion scans of patients were saved as DICOM files and were imported as inputs to our models. rCBV and rCBF parametric files produced by the commercial software were imported the ground truth outputs. Pixel intensities range from 0 to 17199. Higher pixel intensities indicate higher rCBV or rCBF. A data pipeline was built to import and preprocess the data before inputting it into the model. DSC images went through skull stripping and co-registration. The images and the perfusion maps then went through clipping, normalization, and standardization steps. Inputs to the model were separated slice-wise for each subject and were aggregated afterward to generate the final prediction.

### Model Architecture

**Fig 1:**
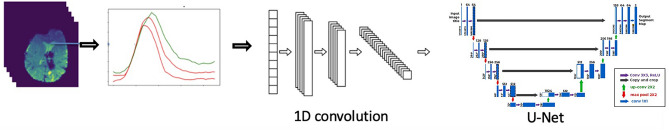
We designed a multistage encoder architecture followed by a decoder to process the MRP datasets. The model captures temporal features followed by a modified U-net to generate the perfusion maps.

We developed a multistage feature extraction and integration model tailored for processing MR Perfusion scans (Figure 1). Our approach begins with a 1D convolutional neural network, serving as the initial encoder, which effectively incorporates information along the time dimension. The features extracted from this CNN encoder are subsequently reshaped and channeled into a 2D encoder-decoder U-Net network. Within the U-Net architecture, a series of down-sampling operations efficiently extract crucial features, followed by a corresponding set of up-sampling steps. These up-sampled features are then intelligently fused with the outputs from the down-sampling layers. The resulting representation is then passed through two additional 2D-CNN layers to derive parametric maps. Ultimately, our model generates predictions that are reassembled into a comprehensive volumetric output, encompassing CBV/CBF estimations for the entire brain volume of the patient. This full dataset, comprising of 60 time-points, was utilized in the training of this model (*model_full*). To explore the importance of temporal features, we adapted from the *model_full* and trained another model (*model_truncated*) with only the first 40 time-points.

### Model Training and Evaluation

The training patient data were randomly divided into two sets: training (70%) and validation (30%). A separate test set containing 28 patients was used as a final evaluation for our best-performing model. In addition to this, we employed a five-fold cross-validation approach to fine-tune our model's hyperparameters. During this process, validation performance metrics were derived as the averages across the 5 distinct validation sets. These averaged metrics guided our hyperparameter grid search, specifically targeting the tuning of the learning rate within the range of 1 × 10^−4^ to 1 × 10^−6^. The models converged after training for 200 epochs. Following fine-tuning with the five-fold cross-validation, we trained a model using the 70-30 training-validation split with our best selected hyperparameters. A final evaluation was conducted on our reserved test set. The models were trained using a single A6000 GPU.

The predicted rCBV and rCBF maps were qualitatively assessed by two neuroradiologists for quality assurance. Each reader evaluated the rCBF and rCBV maps in a randomized order and evaluated them for global image quality and for adequate differentiation between white and gray matter. For quantitative assessment, the mean absolute error (MAE) and root mean square error (RMSE) were calculated using the maps generated by the commercial software as the ground truth.

### Model Baseline

In order to establish a baseline and compare the performance of our model against traditional machine learning methods, we conducted experiments using previous state of the art algorithms, namely Random Forest (RF) and Deep Neural Networks (DNN). These algorithms have been used in previous studies and provide a benchmark to evaluate the effectiveness of our approach [23]. To facilitate training with RF and DNN models, we flattened the voxel data into 1D vectors, enabling these models to process the data effectively.

## Results

After identifying the best-performing model via cross-validation, we conducted a final evaluation on a blind test set comprising 28 patients, which was not used in the model training or cross-validation phases. This approach provided an unbiased assessment of the model's efficacy in a real-world scenario.

Furthermore, to quantitatively characterize the performance of our model, we focused on two metrics: Mean Absolute Error (MAE) and Root Mean Square Error (RMSE). These metrics were calculated based on the pixel value differences between the predicted parametric maps (both rCBV and rCBF) and the corresponding ground truth maps. The use of these two metrics allowed us to capture both the average magnitude of the errors (MAE) and the square root of the average of squared differences (RMSE)

**Fig 2:**
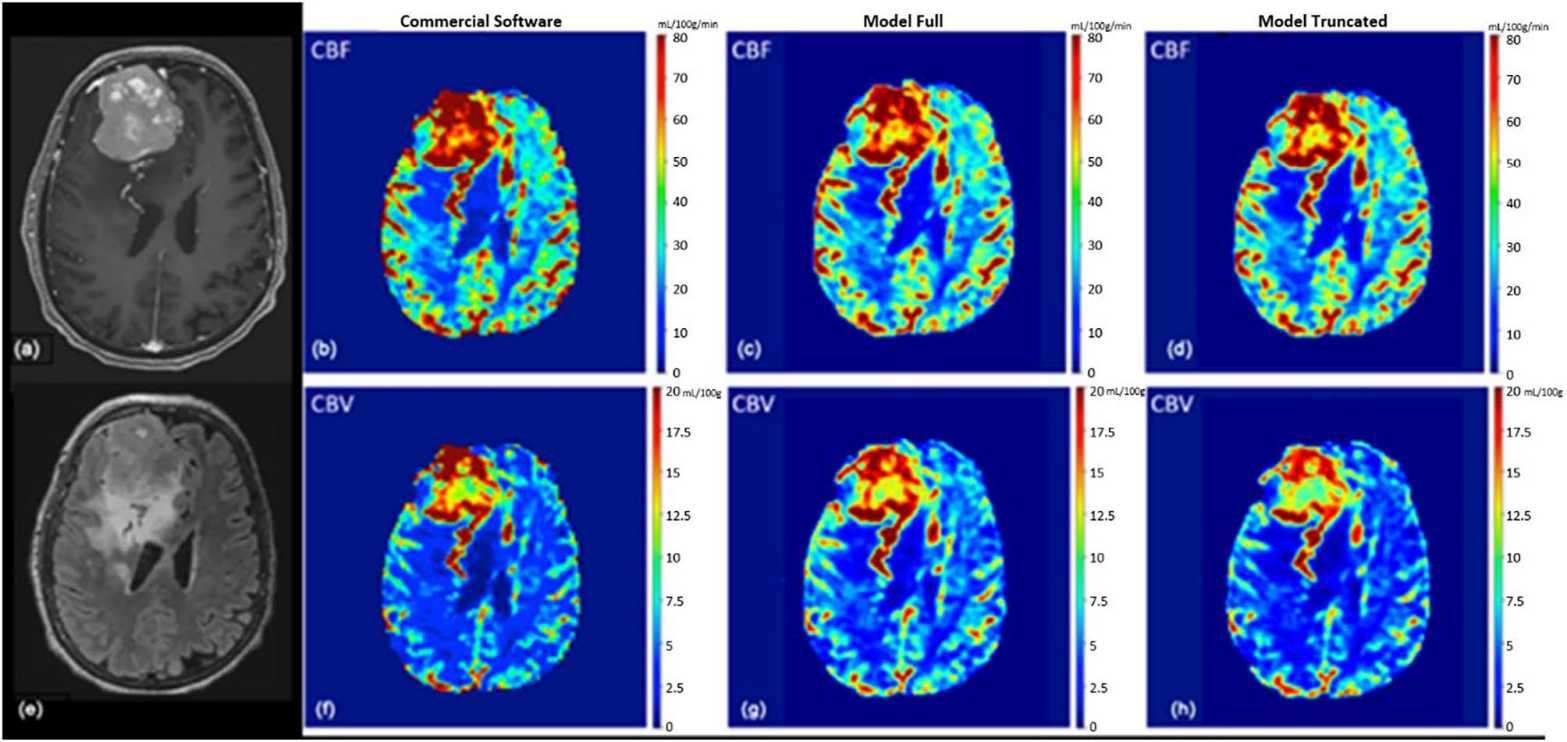
Female in their 80’s with **a** right frontal WHO grade 2 meningioma, measures approximately 5.6 × 6.9 × 4.8 cm with heterogeneous avid enhancement, and surrounding edema. Mass compressed right lateral ventricle and caused midline shift to the left. A few engorged medullary veins developed along the posterior margin of the mass, draining into cortical and medullary veins. **e** There was T2/FLAIR hyperintense edema around the mass. **b** rCBF and **f** rCBV maps calculated by a FDA-approved software. There was markedly elevated rCBF within the mass, and there was elevated rCBV within the mass especially along the anterior and posterior aspects. **c** rCBF and **g** rCBV maps predicted by the model_full, which was trained with 60 time-points. The model_full predicted elevated rCBF within the mass, and elevated rCBV along the anterior and posterior aspects. There were small areas where the predicted rCBF and rCBV were under-estimated. **d** rCBF and **h** rCBV maps predicted by the model_truncated, which was trained with 40 time-points slightly under-estimated the rCBF and rCBF values in a few small areas. **b**, **f** Note, there was also elevated rCBF and rCBV within the engorged medullary draining veins, which were consistently predicted by both model_full and model_truncated. Overall, the predicted rCBV and rCBF maps were diagnostic and useful.

**Fig 3:**
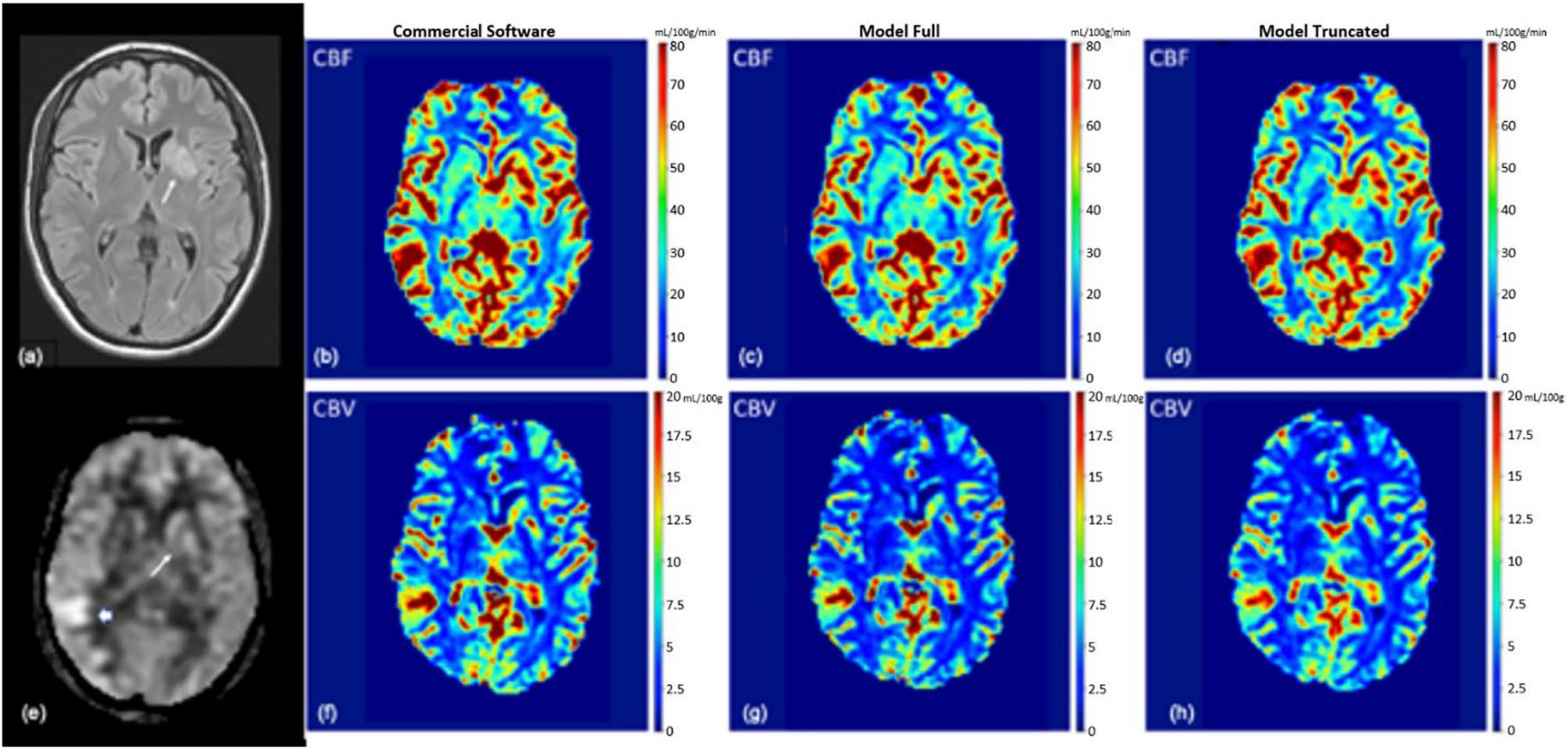
Male in their 20’s presented with seizure with focal neurologic deficits, diagnosed with autoimmune encephalitis. **a** There was a FLAIR T2 hyperintense lesion in left basal ganglia (white arrow). **e** There was associated hyperperfusion in left basal ganglia (white arrow) on ASL (arterial spin labeling). There was also a small area of confluent hyperperfusion in right temporal lobe (thick white arrow) likely related to seizures. **b** rCBF and **f** rCBV maps calculated by a FDA-approved software. There was confluent elevated rCBF within the left basal ganglia and right temporal lobe. There were small areas of elevated rCBV within the left basal ganglia and right temporal lobe. **c** rCBF and **g** rCBV maps predicted by the model_full, which was trained with 60 time-points. The model_full predicted elevated rCBF within the left basal ganglia and right temporal lobe. **d** rCBF and **h** rCBV maps predicted by the model_truncated, under-estimated the rCBF and rCBF values in a few small areas.

The predicted rCBV and rCBF maps were displayed in color and evaluated by two neuroradiologists. Representative results from test subjects are illustrated in Figs. 2 and 3. Qualitatively, all the 28 patients predicted rCBV and rCBF maps from the test set were deemed diagnostic. There were small areas of under-estimated rCBV and rCBF values in the maps predicted by *model_full* and *model_truncated*. There were more under-estimated pixels in the maps predicted by *model_truncated*.

The performance metrics of the *model_full* (trained on 60 time-points) and *model_truncated* (trained on 40 time-points), Random Forest, and Deep Neural Networks from the test set are tabulated in Table 2. For *model_full*, the mean MAE and RMSE for the rCBF maps were 1.91 mL/100 g/min and 7.77 mL/100 g/min, respectively; the mean MAE and RMSE for the rCBV maps were 0.59 mL/100 g and 2.63 mL/100 g, respectively. The performance took a slight hit when the model was trained on fewer time-points. The *model_truncated* scored a slightly lower mean MAE and RMSE for the rCBF maps of 3.11 mL/100 g/min and 10.88 mL/100 g/min, respectively; the mean MAE and RMSE for the rCBV maps were 0.85 mL/100 g and 3.53 mL/100 g, respectively.

**Table 2:** Performance of the *model_full* (trained on 60 time-points), *model_truncated* (trained on 40 time-points), Random Forests, and DNN from the test set

	Model_Full		Model_Truncated		RF		DNN	
	MAE	RMSE	MAE	RMSE	MAE	RMSE	MAE	RMSE
rCBF (mL/100 g/min)	**1.91**	**7.77**	3.11	10.88	4.07	12.76	3.54	12.11
rCBV (mL/100 g)	**0.59**	**2.63**	0.85	3.53	1.04	4.72	0.98	4.03

Bold represents the best-performing model

Our results demonstrate that our spatially aware model outperforms traditional machine learning methods, such as Random Forest (RF) and Deep Neural Networks (DNN), in the task of perfusion parameter estimation. While RF and DNN models are effective at processing flattened 1D voxel data, our model offers a distinct advantage. By leveraging the U-net encoder architecture, our model can capture intricate spatial relationships among neighboring pixels within the image. This spatial context is critical in perfusion imaging, where the flow of contrast agents through the vasculature and tissue is influenced by local interactions.

## Discussion

Dynamic susceptibility contrast magnetic resonance perfusion (DSC-MRP) has a wide range of clinical applications, including the classification of tumors, identification of stroke regions, and characterization of other diseases such as cancer tumors, etc. rCBV and rCBF maps are especially useful in hyper-perfused states, such as epilepsy, tumor, infectious or inflammatory, etc.

Deconvolution is the most frequently used MRP post-processing method, it can be either based on singular value decomposition or Fourier transform [24]. Both post-processing techniques are sensitive to artifacts and noise, and may introduce noise in the estimated output parametric maps. Deconvolution methods require the selection of two vascular waveforms—the arterial input function (AIF) and the venous output function.

There is no consensus in the optimal choice of AIF and VOF. The time-intensity waveform of the AIF can differ based on the artery selected, as a result, the parametric maps may vary according to the selection of AIF. Similarly, the parametric maps may also vary according to the choice of VOF. When performed manually, the choice of AIF and VOF can be affected by noise and operator’s experience. Poor reproducibility of vessel selection will result in unreliable perfusion maps. It is, therefore, desirable to develop a method that is robust to noise and the choice of vascular functions.

In recent years, deep learning (DL) models have been shown to be a valuable technique in radiology. Besides using DL for detection and segmentation intracranial abnormalities in brain images [25, 26], there are also DL models for predictions from input images [27, 28]. DL methods have also been used in MRP. There are commercial MRP software that have built-in automated AIF and VOF selection tools that utilize DL [29]. Although the underlying algorithms of most the commercial software are proprietary, other groups have published their algorithm [30–32]. Using DL methods to select the AIF and VOF were more reproducible and less operator-dependent. Once AIF and VOF were selected, the perfusion parameters are calculated by conventional convolution methods. As such, DL is only limited to the selection of vascular input functions. McKinley et al. took this further and used DL to generate perfusion maps by comparing the voxel-wise intensity-time waveform with the pre-selected AIF waveform. They compared several machine learning techniques such as random forests, linear regression, and neural network to generate the perfusion parametric maps. Our approach does not require pre-selection of vascular input or output functions. Instead, our model implicitly incorporates the arterial input waveforms and venous output waveforms as inherent variables embedded within each 4D-dataset.

Traditional perfusion post-processing operates on a voxel level. Information from neighboring voxels may be incorporated by applying a spatial smoothing filter before the deconvolution step. In our approach, temporal features and spatial information are very purposefully and deliberately combined together to generate the perfusion maps. In our two-step encoder approach, temporal features are first extracted from each voxel. In the second stage, these temporal features are then integrated with spatial features through a U-Net neural network. To illustrate the importance of the foremost step of temporal feature extraction, we trained a model, with similar architecture, with truncated datasets. As expected, this resulted in a small hit in the performance of the model. Conventionally, cerebral blood volume is obtained by integrating the area under the deconvolved tissue concentration-time curve [33]. A ML model trained with truncated waveform will likely produce less accurate results. Similarly, cerebral blood flow, which is estimated by the slope of the deconvolved tissue concentration-versus-time curve, will also be impacted by shortened waveforms. Our findings confirm that temporal features of the intensity-time waveforms have important impact on the overall performance.

Deep learning architectures have demonstrated their utility as an effective strategy for generating parametric maps from perfusion scans. Further testing in real-life settings will be necessary to validate the accuracy and usability of the DL-based perfusion maps. Once validated, this model can be deployed on workstations used by radiologists to process the perfusion datasets. Parametric maps can be generated within seconds and displayed on the monitors, or they can be stored as images within the radiology archiving system. By generating perfusion maps while considering all pixels and eliminating the need for the selection of AIF and VOF, the variability of the perfusion parameters induced by the choice of AIF/VOF is reduced. This approach may hypothetically reduce the variability among different software vendors. Additionally, the prohibitive cost of many available commercial software vendors is limiting the availability of perfusion imaging in many small or rural hospitals and imaging centers. This DL-based method may act as an affordable and dependable alternative for generating perfusion parametric maps for the assessment of acute ischemic stroke, brain tumors, and other diseases.

Furthermore, our current model demonstrates comparable results to those obtained from FDA-approved commercial software. Quantitatively, our model's performance aligns with ground truth maps, while qualitatively, it produces rCBV and rCBF maps suitable for clinical assessment by radiologists. The model adeptly delineates pathologies, albeit with slight underestimations in absolute values, largely attributable to the size of our training set. While these minor discrepancies do not compromise diagnostic quality, they warrant further fine-tuning to enhance quantitative precision.

### Limitations

Our study has several limitations. The generalizability of the model is limited since our data came from a single center. Therefore, further studies with multicenter data will be needed to evaluate how susceptible our model is to distribution shift. Larger number of datasets may also improve the performance.

## Data Availability

The datasets utilized during this study are not publicly available due to reasonable privacy and security concerns. The data are not easily redistributable to researchers other than those engaged in the Institutional Review Board-approved research collaborations with Stanford University.
